# Optimality of multisensory integration while compensating for uncertain visual target information with artificial vibrotactile cues during reach planning

**DOI:** 10.1186/s12984-024-01448-0

**Published:** 2024-09-09

**Authors:** Lukas K. Amann, Virginia Casasnovas, Jannis Hainke, Alexander Gail

**Affiliations:** 1 Faculty of Biology and Psychology, Georg-August University, Goßlerstr. 14, 37073 Göttingen, Germany; 2 Sensorimotor Group, German Primate Center, Kellnerweg 4, 37077 Göttingen, Germany; 3 Bernstein Center of Computational Neuroscience, Heinrich-Düker-Weg 12, 37077 Göttingen, Germany; 4 Leibniz ScienceCampus Primate Cognition, Kellnerweg 4, 37077 Göttingen, Germany

**Keywords:** Sensory augmentation, Vibrotactile stimulation, Multisensory integration, Reaching

## Abstract

**Background:**

Planning and executing movements requires the integration of different sensory modalities, such as vision and proprioception. However, neurological diseases like stroke can lead to full or partial loss of proprioception, resulting in impaired movements. Recent advances focused on providing additional sensory feedback to patients to compensate for the sensory loss, proving vibrotactile stimulation to be a viable option as it is inexpensive and easy to implement. Here, we test how such vibrotactile information can be integrated with visual signals to estimate the spatial location of a reach target.

**Methods:**

We used a center-out reach paradigm with 31 healthy human participants to investigate how artificial vibrotactile stimulation can be integrated with visual-spatial cues indicating target location. Specifically, we provided multisite vibrotactile stimulation to the moving dominant arm using eccentric rotating mass (ERM) motors. As the integration of inputs across multiple sensory modalities becomes especially relevant when one of them is uncertain, we additionally modulated the reliability of visual cues. We then compared the weighing of vibrotactile and visual inputs as a function of visual uncertainty to predictions from the maximum likelihood estimation (MLE) framework to decide if participants achieve quasi-optimal integration.

**Results:**

Our results show that participants could estimate target locations based on vibrotactile instructions. After short training, combined visual and vibrotactile cues led to higher hit rates and reduced reach errors when visual cues were uncertain. Additionally, we observed lower reaction times in trials with low visual uncertainty when vibrotactile stimulation was present. Using MLE predictions, we found that integration of vibrotactile and visual cues followed optimal integration when vibrotactile cues required the detection of one or two active motors. However, if estimating the location of a target required discriminating the intensities of two cues, integration violated MLE predictions.

**Conclusion:**

We conclude that participants can quickly learn to integrate visual and artificial vibrotactile information. Therefore, using additional vibrotactile stimulation may serve as a promising way to improve rehabilitation or the control of prosthetic devices by patients suffering loss of proprioception.

**Supplementary Information:**

The online version contains supplementary material available at 10.1186/s12984-024-01448-0.

## Background

Information from different sensory modalities must be integrated to control and execute movements properly. During reaching movements, the visual system provides information about the location of potential movement goals and the hand. At the same time, proprioceptive signals are required to provide information about the state of muscles and joints [1, 2]. However, various neurological conditions can lead to a full or partial loss of proprioception or impaired processing [3–5], which leads to impaired movement control [6]. For instance, proprioceptive deficits are common in stroke patients [7], affecting control of upper limbs [8, 9] since, for many stroke survivors, vision cannot fully compensate for the impaired position sense [10]. Recently, research focused on providing additional artificial feedback to patients in other sensory modalities to compensate for the lack of proprioception [11, 12] and providing supplementary sensory feedback to improve the control of neuro-prostheses and brain-computer interfaces, where no proprioceptive inputs are available [13–17]. One inexpensive way of delivering such supplementary feedback is vibrotactile stimulation on the skin. However, how humans integrate such vibrotactile information with visual signals is still unclear.

It has been shown that humans can use pure vibrotactile input in different ways to infer extrapersonal position information. Risi et al. [18] showed that human participants could control movements based on vibrotactile stimulation to their contralateral, non-moving arm without visual feedback. The authors provided vibrotactile information about the current position of the moving hand, that is, state feedback. Alternatively, information about the motor error could be given, namely the spatial distance between hand position and reach goal. Availability of error feedback has been shown to result in higher performance [19]. Additionally, Ballardini et al. [20] provided evidence that stroke survivors can use vibrotactile state or error feedback to guide their movements.

These previous studies demonstrate the usefulness of vibrotactile feedback in the absence of visual feedback. Yet, able-sighted patients can also make use of vision to localize target objects and the hand. While this is true in good light conditions in the visual part of their workspace, vision is less helpful in low-light conditions, when working in the periphery of the visual field or behind the back or an occluding object. Cross-modal calibration and flexible weighing of visual and somatosensory information is therefore important to allow smooth acting in situations of varying access to visual information to arrive at a coherent percept. Combining multiple sources of information becomes especially beneficial if one or more inputs are uncertain [21]. Therefore, we want to test if and how participants can learn to integrate vibrotactile and uncertain visual inputs when both provide spatially congruent information.

The way humans integrate inputs from multiple sensory modalities is mostly conceptualized in the maximum likelihood estimation (MLE) framework (for review see 22). According to this theory, modalities are integrated by summing their unimodal estimates weighted by their respective variances. This means that the more reliable input is assigned a greater weight. Since even less reliable inputs carry some information, albeit with higher uncertainty, it is still beneficial to consider these inputs in the overall integration process. Integrating multisensory inputs in this optimal statistical manner results in a more precise (i.e., less variable) and more accurate bimodal estimate. MLE integration has been demonstrated across various sensory systems, such as visual-haptic integration [23], audiovisual signals [24, 25], texture and motion cues [26], and olfactory-visual signals during the perception of emotions [27]. However, studies also found examples of suboptimal integration [28–30] or raised caution towards over-interpreting previous results demonstrating MLE integration [31, 32].

In this study, we wanted to test if healthy participants (i) can use vibrotactile information to estimate the location of a reach target correctly, (ii) use congruent vibrotactile information to compensate for uncertain visual inputs, and (iii) integrate visual and vibrotactile inputs following MLE predictions. We provided participants with visual and artificial multisite vibrotactile cues about the location of reach targets. We used a discretized vibrotactile stimulation protocol and applied the vibration using four eccentric-rotating mass (ERM) motors attached to the moving arm. We varied visual cue reliability to simulate uncertain sensory inputs and to encourage integration. Insights gained from this study may inform the development of novel rehabilitation strategies for individuals with proprioceptive deficits and the design of more intuitive and responsive neuro-prostheses and computer interfaces.

## Methods

### Participants

We tested 32 participants with no prior experience in cutaneous vibrotactile stimulation for sensory discrimination tasks. One participant was excluded because the minimum performance criterion during training was not reached, leaving 31 participants for analysis (17 female, 24.8 ± 4 [s.d.] years). All participants self-identified as right-handed, had normal or corrected-to-normal vision, and reported no neurological or muscular deficits. Each participant attended two sessions on separate days within one week, lasting approximately 1 h, and received financial compensation for their participation (8 €/h). Before the experiments, participants gave written informed consent. The study was conducted following institutional guidelines for experiments with humans, adhered to the principles of the Declaration of Helsinki, and was approved by the Ethics Committee of the Georg-Elias-Mueller-Institute for Psychology at the University of Göttingen.

### Experimental setup

**Fig. 1:**
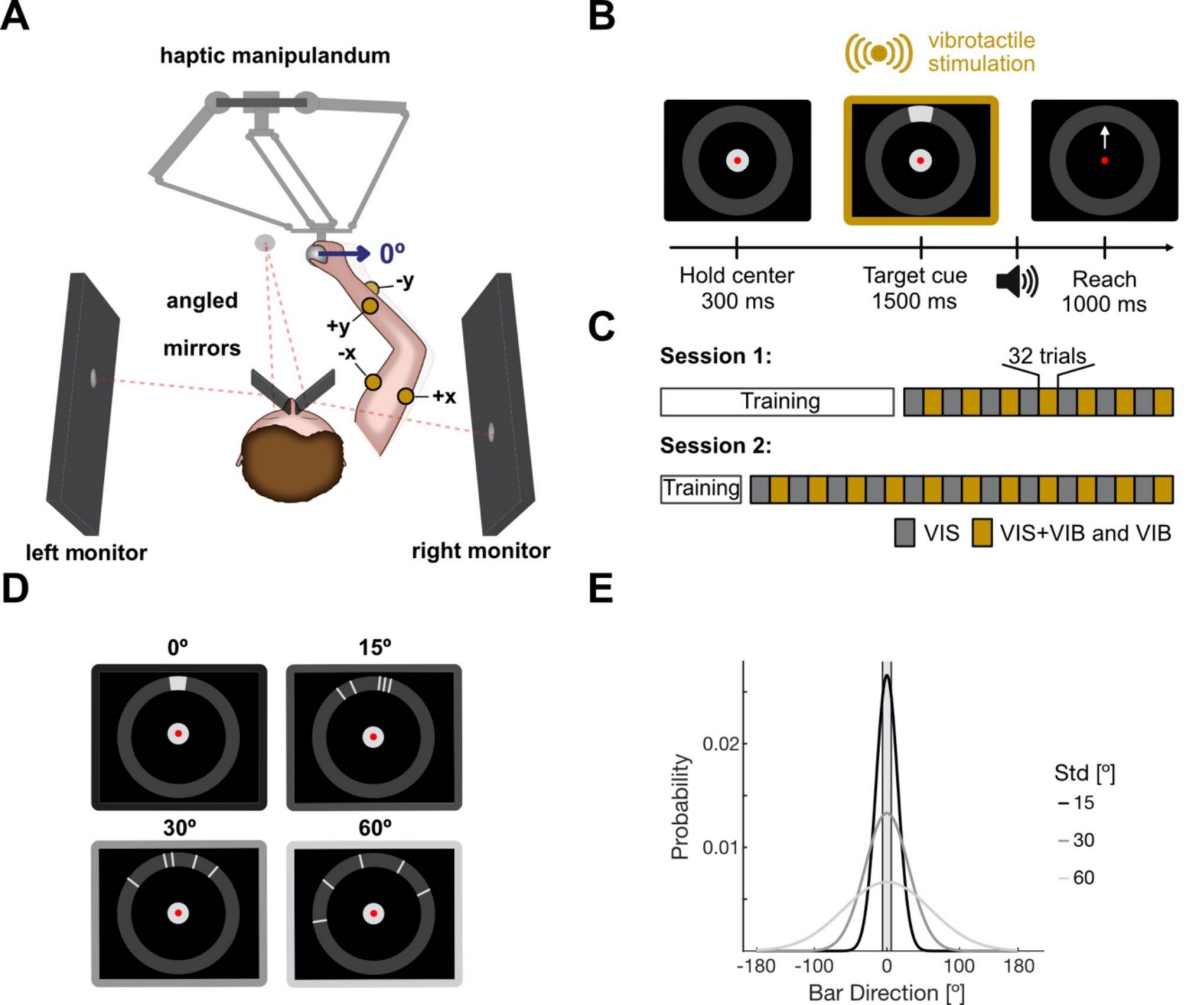
Experimental setup and task design. **(A)** Participants are seated in front of a haptic manipulandum performing center-out reaches. The image of two angled monitors is mirrored in front of the participant. Four ERM motors (yellow circles) are placed on the moving arm, each encoding one cardinal direction. Movements to the right were considered to be in a direction of 0° (blue arrow). **(B)** Task design. A trial started with a holding period of 300 ms in which the cursor (red dot) was moved into a central fixation point (light gray circle). Next, targets at one of 16 possible directions along the target ring were cued for 1500 ms using either visual (VIS), vibrotactile (VIB), or bimodal cues (VIS + VIB). Participants had 1000 ms to initiate their movement and leave the fixation point area, after fixation point disappeared and Go sound was played. The outer target ring needed to be reached within 1000 ms after leaving the fixation point, causing the trial to end. Yellow frame highlights the period in which motors were active. **(C)** Block design. Cue modality was switched between blocks of 32 trials. Blocks with only VIS trials in gray and with VIS + VIB and VIB trials in yellow. All participants completed 36 blocks over two sessions (14 in session 1 and 22 in session 2). **(D)** Example illustration of visual cues with different levels of target uncertainty. Either the target location was revealed (0°) or the target was cued using five bars. Their position was drawn from a normal distribution centered around the target location. **(E)** Distributions for different uncertainty levels (15°, 30°, 60°). The shaded area resembles target size

Participants were seated comfortably in a darkened room within an augmented reality setup [33] using a haptic manipulator (delta.3, Force Dimension) to perform reaching movements in a 2D plane (Fig. 1A). The image of 2 monitors (27-inch diagonal, 60 Hz refresh rate, XL2720T, BenQ) on either side of the participant (viewing distance 47 mm) was projected in front of the participants using two semi-transparent mirrors (75 × 75 mm, stock #46–643, Edmund Optics Inc.) angled 45° relative to the monitors. We covered the back of the mirrors with black cardboard to prevent participants from seeing their moving hands.

A computer running custom software (C++, OpenGL) controlled the experimental task, visual stimulus generation, and hand position recording (sampled at 2 kHz). The computer was connected to a microcontroller (Teensy 3.5, PJRC) that controlled four eccentric-rotating mass (ERM) vibration motors (310-003, Precision Microdrives) via haptic controllers (DRV2605L, Adafruit) and a multiplexer (TCA9548A, Fasizi) for vibrotactile stimulation.

### Experimental paradigm

To test how well participants can integrate vibrotactile reach instructions when the visual target location is uncertain, participants performed center-out reaches within a 2D workspace using the haptic manipulandum. The testing phase for the 2 sessions was the same for all participants: At the beginning of a trial, they were instructed to fixate a gray circle (10 mm radius) positioned in the workspace’s center (Fig. 1B). The fixation point was surrounded by a dark grey ring (inner radius 90 mm, outer radius 110 mm) in which possible targets are located. Following a brief hold period of 300 ms, a target (20 mm arc, 100 mm distance to fixation point) located at one of 16 locations was cued for 1500 ms (4 cardinal, 4 oblique, 8 intermediate). Following the disappearance of the central fixation point and a simultaneous auditory signal, participants had 1000 ms to initiate their movement and leave the area of the fixation point (max. reaction time). Afterwards, they had another 1000 ms to reach the estimated target location (max. movement time). They were asked to aim for the target as accurately as possible. No movement speed constraint other than the time limit was applied. When the cursor reached the outer ring, the trial ended. No feedback about the success of the trial or the actual target location was provided. This was done to prevent participants from continuing to learn the vibrotactile mapping throughout the testing phase of the experiment. After 300 ms, the fixation point reappeared and the next trial was started by moving the cursor into its center.

Targets were cued either visually (VIS), by vibrotactile stimulation (VIB), or with a combination of both (VIS + VIB). Cue modalities were switched in alternating blocks of 32 trials and indicated to the participant by the cursor’s color (Fig. 1C) to ensure participants were paying attention to the relevant sensory inputs. A yellow cursor instructed participants that targets would only be cued visually, whereas a red cursor indicated VIB or VIS + VIB trials. All participants completed a total of 1152 trials (36 blocks). Within each block, target direction and uncertainty level were randomly interleaved. Participants completed 14 blocks on the first day and 22 on the second.

Additionally, visual target uncertainty was varied by target cues consisting of a cloud of bars distributed along the ring (Fig. 1D) [34]. The position of each bar was drawn from a probability distribution centered on the target location. For target uncertainty trials, we used distributions with three different standard deviations (15°, 30°, 60°) to modulate the spread of the bars (Fig. 1E). Within each modality block, uncertainty levels were randomly interleaved. Visual target cues were always well visible (high contrast), the target uncertainty resulted purely from the spatial configuration. Also, the size of the target relevant for task performance did not change with uncertainty but was always an arc of 20 mm length.

### Vibrotactile stimulation

To provide vibrotactile reach instructions to the participants, we placed four ERM motors on their actively moving right arm using self-adhesive tape. The ERM motors have an operational frequency range of 50–190 Hz and an amplitude of 0.6–1.1 g. Vibration frequency and amplitude of ERM motors co-vary, which has been shown to be beneficial for the perception of vibration [35]. Two motors were placed at the initial third of the lower arm, situated from the wrist, while the other two were attached to either side of the biceps muscle (Fig. 1A). Each motor encoded a target in one of the four cardinal directions: The motors on the lower arm cued targets located up (90°) or downwards (270°) and the motors on the biceps encoded targets to the left (180°) or right (0°) of the center. To encode the oblique (45°, 135°, 225°, and 315°) directions, two motors were activated simultaneously with the same intensity. To encode the intermediate directions (22.5°, 67.5°, etc.), two motors were activated with asymmetric intensities.

More specifically, when a target was located in either a cardinal or oblique direction, the corresponding motor(s) were fully active, generating a vibration frequency of approximately 190 Hz and an amplitude of about 1.1 g. In the case of a target located in an intermediate direction, the motor encoding a cardinal direction closest to this target was fully activated, while the more distant motor operated at a lower intensity, using a vibration frequency of approximately 120 Hz and an amplitude of about 0.4 g. We note that frequency and amplitude values can only be approximated since they depend on the attached mass. While we tried to reproduce as well as we could the pressure with which the motors were attached to the arm between participants and sessions, a certain variability was unavoidable. Variability in pressure can translate into slight differences in the motor amplitudes.

### Training

Each participant took part in a training session on the first day to familiarize themselves with vibrotactile stimulation and the experimental setup. During training, we introduced vibrotactile stimulation alongside visual cues. The training consisted of three blocks: in the first block, targets located along the cardinal axis were cued. In the second block, targets along the oblique directions were added, followed by the third block in which targets could appear in all 16 possible directions.

Each block consisted of two parts: in the first part, targets were cued visually and vibrotactilely, and participants were instructed to reach the target. After each target of that block was presented at least three times, participants advanced to the second part of the given block if the hit rate in the last ten trials exceeded 80%. In the second part, targets were cued exclusively through vibrotactile stimulation. Participants needed to achieve the same performance criterion to progress to the next block. Participants were informed about the target location at the end of a trial throughout the training in case they missed it. In the last block with all 16 target directions, we applied the performance criterion only to the first part with bimodal cues. This was necessary since most participants could not reach the required hit rate based on vibrotactile cues alone. In that case, training was stopped after 30 to 40 min of total training time, depending on how quickly the participant completed the previous blocks. On average, participants performed 27.63 ± 2.65 (SEM) trials for each target in cardinal directions, 22.97 ± 2.64 in oblique directions, and 18.71 ± 3.14 trials for each target in intermediate directions. On the second day, the second block with 8 directions was repeated to remind participants of the stimulation protocol.

### Data analysis

Data were stored for offline analysis with MATLAB^®^ R2021a (MathWorks Inc.). All plots were generated with GRAMM visualization toolbox for MATLAB [36]. Movement trajectories were aligned to movement onset, defined as the time at which the hand velocity exceeded 0.02 mm/ms. For analysis, we excluded all trials where participants did not enter the target ring at a minimum eccentricity of 90 mm (1.4% of all trials). The hit rate was defined as the percentage of trials in which the cursor ended within the target arc. The mid-reach error was calculated by computing the absolute angular difference of the cued target direction and the vector pointing from the fixation point to the cursor after traveling half the distance to the target ring. To compute mid-reach standard deviations, we rotated all trajectories within a given uncertainty and cue modality, irrespective of the target direction, to a direction of 0°. We then calculated the circular variance and standard deviation across all trials of the given condition using the CircStat toolbox for MATLAB [37]. Reaction time was defined as the time between Go-cue and movement onset while movement time refers to the time from movement onset until reaching the outer ring.

### Optimal integration predictions

Bayesian integration theory describes the integration of two noisy sensory inputs into one bimodal estimate. Here, the estimated bimodal target location for a given visual target uncertainty level $\:{S}_{VIS+VIB,u}$ can be derived from the weighted sum of visual and vibrotactile estimated target locations $\:{S}_{VIS,u}$ and $\:{S}_{VIB}$:

1 $$\:{S}_{VIS+VIB,u}={w}_{VIS,u}{S}_{VIS,u}+{w}_{VIB,u}{S}_{VIB}$$

with weights being proportional to the unimodal variances:

2 $$\:{w}_{VIS,u,mle}=\frac{{\sigma\:}_{VIS,u}^{-2}}{{\sigma\:}_{VIS,u}^{-2}+{\sigma\:}_{VIB}^{-2}};{\:w}_{VIB,u,mle}=\frac{{\sigma\:}_{VIB}^{-2}}{{\sigma\:}_{VIS,u}^{-2}+{\sigma\:}_{VIB}^{-2}}$$

Here, only the variance of the visual estimate is dependent on the uncertainty level since we did not experimentally manipulate the uncertainty of the vibrotactile cues and, therefore, assume it is constant across uncertainty levels. The bimodal variance for a given uncertainty level $\:{\sigma\:}_{VIS+VIB,u}^{2}$ is given by:

3 $$\:{\sigma\:}_{VIS+VIB,u}^{2}=\frac{{\sigma\:}_{VIS,u}^{2}{\sigma\:}_{VIB}^{2}}{{\sigma\:}_{VIS,u}^{2}+{\sigma\:}_{VIB}^{2}}$$

Equation 3 describes the reduced variance of the bimodal estimate compared to any of the unimodal variances, which is necessarily fulfilled in the case of optimal cue integration. We compared the variance of the mid-reach angle in VIS + VIB trials to the variance in the corresponding VIS or VIB trials, depending on which was lower, to test this prediction for each subject and uncertainty level.

Next, we compared our data to predicted values from the MLE framework. Given that motor variability can also influence the observed behavioral variance, we assumed it would be reflected in the smallest reach variance $\:{\sigma\:}_{MIN}^{2}\:$across conditions [38]. From that, we determined the predicted bimodal variance for a given uncertainty level as follows:

4 $$\:{\sigma\:}_{VIS+VIB,u,mle}^{2}=\frac{{(\sigma\:}_{VIS,u}^{2}-{\sigma\:}_{MIN}^{2}){(\sigma\:}_{VIB}^{2}-{\sigma\:}_{MIN}^{2})}{{\sigma\:}_{VIS,u}^{2}+{\sigma\:}_{VIB}^{2}-{2\sigma\:}_{MIN}^{2}}+{\sigma\:}_{MIN}^{2}$$

To compare the predicted visual weights $\:{w}_{VIS,mle,u}$ (Eq. 2) with our data, we calculated the empirical visual weights for each uncertainty level and subject by:

5 $$\:{w}_{VIS,emp,u}=\frac{{\sigma\:}_{VIB}^{2}-{\sigma\:}_{VIS+VIB,u}^{2}}{{\sigma\:}_{VIB}^{2}}$$

### Statistics

We fitted a series of generalized linear mixed models (GLMM) in R (version 4.2.3.) to determine the effect of target uncertainty and cue modality on hit rate (model 1), mid-reach error (model 2), reaction time (model 3), and movement time (model 4) using the package ‘glmmTMB’ (version 1.1.8) [39]. We used the package ‘emmeans’ (version 1.9.0) for post-hoc testing [40] on the models’ estimated marginal means (EMMs). Specifically, we used paired z-tests since the variance derived from the model is known, and the sample size is large because all trials were included in the models. We included uncertainty level, cue modality, and their interaction as fixed effects and added random slopes and intercepts for each participant to account for repeated trials. Models were fitted to all VIS and VIS + VIB trials, excluding VIB trials, since we wanted to test for improvements in the bimodal condition over VIS trials. We included uncertainty level as a factor since we expected its effect to be non-linear. Note that using factors results in *n* – 1 coding variables, where *n* is the number of levels, to avoid singularities of the design matrices of the models. We did not include estimation of the correlation parameters between random intercepts and slopes. Model 1 was fitted with binomial error distribution and logit link to model the hit rates. To fit mid-reach errors, we transformed them to a proportion ([0, 1]) from the range of possible reach errors ([0°, 180°]) and fitted model 2 with beta error distribution and logit link [41, 42]. We similarly transformed reaction times and movement times to a proportion of the possible range ([0 ms, 1000 ms]). Next, we fitted models 3 and 4 with beta error distribution and logit link function.

Additionally, we used the same method to compare the data with the MLE predictions. First, we fitted a GLMM to the mid-reach circular variance in VIS + VIB trials and the corresponding minimum unimodal variance (model 5). We used uncertainty level and type (minimum unimodal or bimodal variance) as fixed effects and included the same random effects as in the previous models. Next, to compare our data to MLE predictions, we fitted a GLMM to mid-reach circular variance (model 6) or visual weight (model 7). In those models, type differentiated between observed data and MLE prediction. Since circular variance and visual weights are defined in a range from 0 to 1, we again fitted models 5 to 7 with beta error distributions and logit link function.

Models with the additional letter *a* were fitted to the whole dataset, while models with the additional letter *b* were fitted to trials with only cardinal and oblique targets. We first compared all full models to a reduced model without the effect of either cue (model 1 to 4) or type (model 5 to 7). If likelihood-ratio tests showed significantly better fits, the following analyses were performed using the full model. For all models, we determined the significance of individual effects by dropping them from the model, one at a time, and comparing the likelihood of the resulting models with that of the full model (R function *drop1*). We estimated 95% confidence limits of model estimates and fitted values using parametric bootstraps (*N* = 1000 bootstraps; function *simulate* of the package ‘glmmTMB’). Finally, we evaluated the stability of the models on the estimated coefficients and standard deviations by excluding each subject once, fitting the full model to each of the subsets, and comparing the obtained range of estimates to those from the full dataset. All models showed good stability.

For simple comparisons between VIS trials with 60° visual uncertainty and VIB trials, we used a paired t-test on participants’ means after testing for normality of the data. We considered p-values smaller than 0.05 significant. P-values of post-hoc tests were adjusted for multiple comparisons using the Bonferroni-Holm method.

## Results

### Estimation of target location using vibrotactile cues

**Fig. 2:**
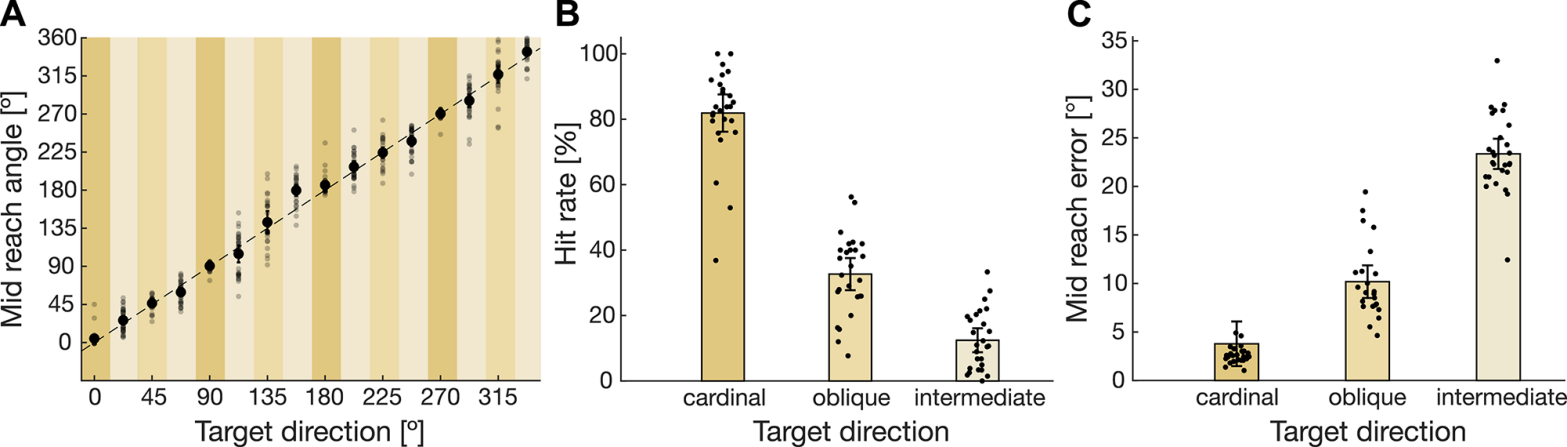
Performance in VIB trials. **(A)** Mid-reach angle compared to actual target location across all participants (*N* = 31). Small points represent individual participants. Shaded areas highlight cardinal (dark), oblique (medium), and intermediate directions (bright). **(B)** Hit rate for different target directions across all participants. Points represent single participants. **(C)** Mid-reach error across all participants. Error bars depict 95% confidence intervals

First, we tested whether participants could accurately estimate a target’s location using vibrotactile cues alone. The mid-reach angles matched the actual target direction (Fig. 2A), demonstrating that participants were indeed able to use vibrotactile cues to estimate the location of a target. Additionally, we found some variability across targets, displaying more errors for oblique and especially for intermediate directions. To further investigate these differences, we calculated the performance across participants depending on the target directions (Fig. 2B). We found that the hit rate associated with cardinal directions was higher compared to oblique and intermediate directions in VIB trials. Additionally, mid-reach errors were lower for reaches towards cardinal targets compared to other target directions (Fig. 2C).

### Effect of bimodal cues on task performance

**Fig. 3:**
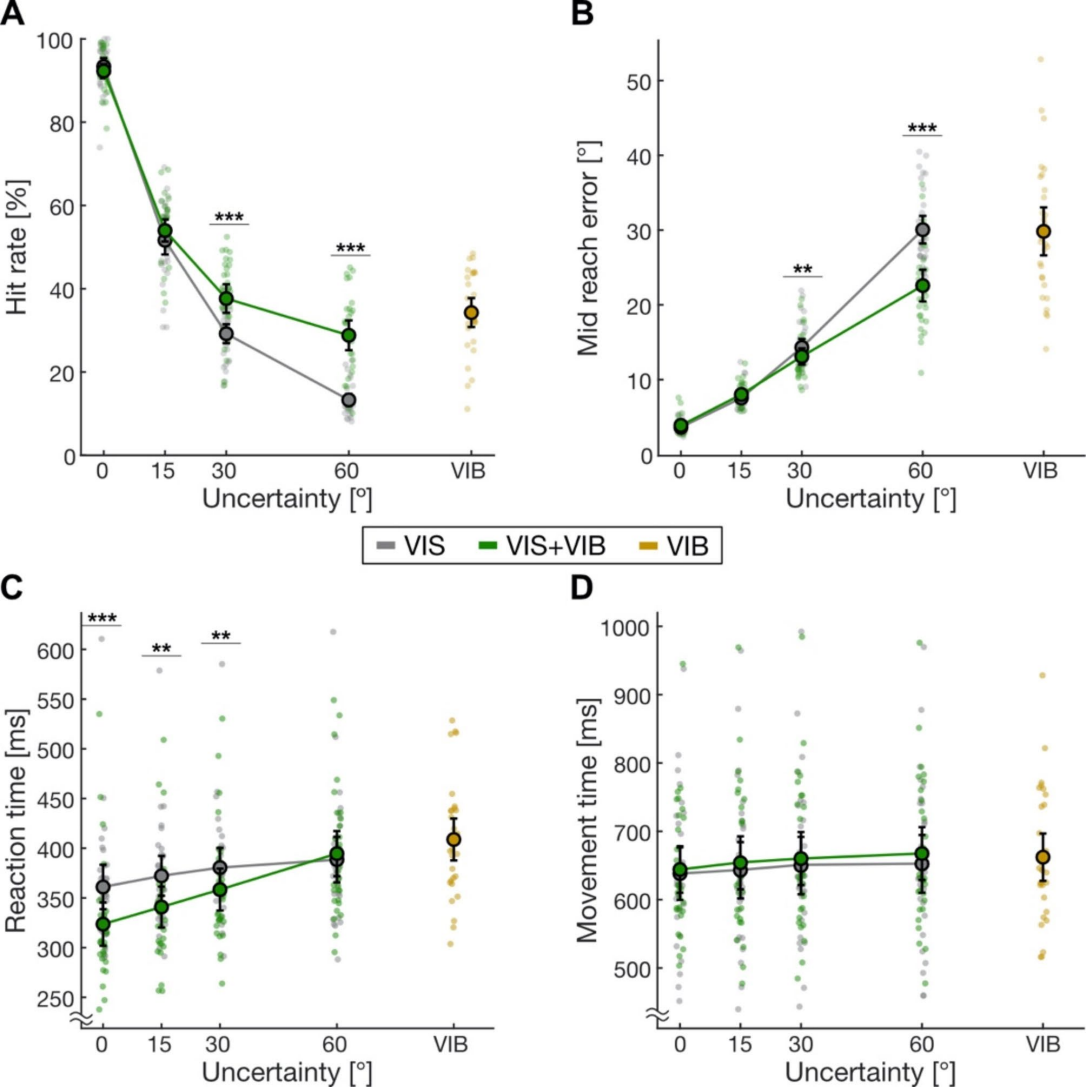
Participants’ performance dependent on uncertainty level and cue modality. **(A)** Mean hit rate. **(B)** Mean mid-reach error. **(C)** Mean reaction time. **(D)** Mean movement time. Results from VIS trials in grey, VIS + VIB trials in green, and VIB-only trials in yellow. Shaded points show data from individual participants (*N* = 31). Error bars depict 95% confidence intervals. *p < 0.05, **p < 0.01, ***p < 0.001 for paired z-tests

We first tested if the overall performance of participants improved with additional vibrotactile cues (Fig. 3A). Fitting model 1a to the hit rates, we found that the full model fit the data significantly better than the reduced model (likelihood-ratio test, χ^2^_(4)_ = 99.95, *p* < 0.001). Using the full model, we found a significant interaction between uncertainty level and cue modality (likelihood-ratio test, χ^2^_(3)_ = 75.17, *p* < 0.001). Post-hoc tests revealed significantly higher hit rates in VIS + VIB trials when visual target uncertainty was high (paired z-test, 30°: z = − 7.44, *p* < 0.001; 60°: z = − 13.06, *p* < 0.001). There was no significant difference for low (0°, 15°) levels of uncertainty (paired z-test, 0°: z = 1.88, *p* = 0.06; 15°: z = − 1.67, *p* = 0.09). Participants’ hit rates in VIB trials were higher compared to VIS trials at an uncertainty level of 60° (paired t-test, t_(30)_ = − 13.93, *p* < 0.001). These findings demonstrate that participants benefitted from vibrotactile cues.

Since the hit rate is a binary measure and can only give limited insights into participants’ accuracy in estimating the target location, we compared the absolute mid-reach error across uncertainty levels and cue modalities (Fig. 3B). Again, we found that the full model (model 2a) fit the data significantly better than the reduced model ((likelihood-ratio test, χ^2^_(4)_ = 110.0, *p* < 0.001). Results from the full model revealed a significant interaction of uncertainty level and cue modality, as seen before for the hit rate (likelihood-ratio test, χ^2^_(3)_ = 77.52, *p* < 0.001). Using post-hoc tests, we found that mid-reach errors were significantly lower in VIS compared to VIS + VIB trials at 30° and 60° uncertainty (paired z-test, 30°: z = 5.09, *p* < 0.001; 60°: z = 15.06, *p* < 0.001), whereas there was no significant difference for low uncertainty levels (paired z-test, 0°: z = − 0.24, *p* = 0.80; 15°: z = 0.38, *p* = 0.70). Reach errors in VIS trials at the highest uncertainty level were similar to mid-reach errors in VIB trials (paired t-test, t_(30)_ = 1.03, *p* = 0.31).

Finally, we wanted to test if the planning or the execution of the movements are affected by target uncertainty and during which time integrating additional vibrotactile information can have a benefit. Therefore, we analyzed reaction and movement times (Fig. 3C, D). For reaction times, the full model 3a fit the data significantly better (likelihood-ratio test, χ^2^_(4)_ = 56.45, *p* < 0.001). In contrast, there was no difference between the full and reduced model (model 4a) for the movement times (likelihood-ratio test, χ^2^_(4)_ = 8.64, *p* = 0.07). Model 3 results revealed a significant interaction of uncertainty level and cue modality for the reaction times (likelihood-ratio test, χ^2^_(3)_ = 47.80, *p* < 0.001). Still, we did not find a significant effect of cue modality on movement times since it was not included in the reduced model. In addition to benefits in accuracy in trials with high uncertainty, we found lower reaction times for VIS + VIB trials compared to VIS trials for low levels of uncertainty (paired z-test, 0°: z = 5.39, *p* < 0.001; 15°: z = 5.23, *p* < 0.001; 30°: z = 5.12, *p* < 0.001; 60°: z = 0.92, *p* = 0.36). Detailed GLMM results for all models can be found in Table S1.

### Integration of visual and vibrotactile cues

**Fig. 4:**
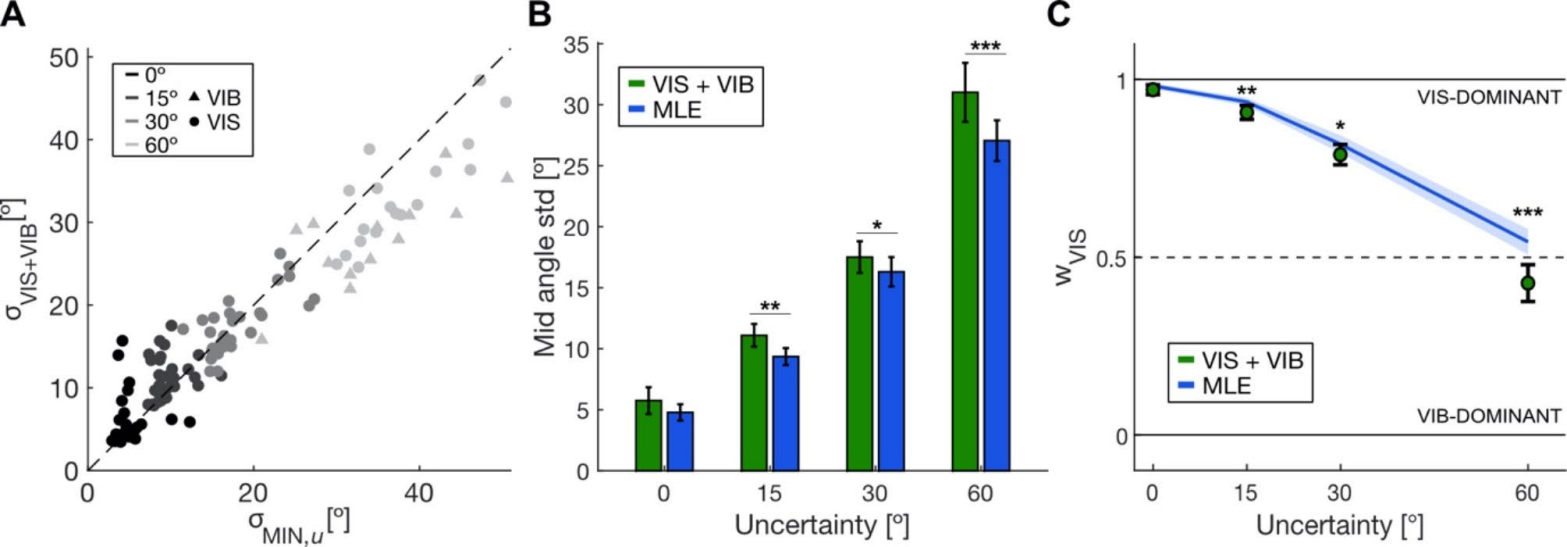
MLE predictions including all directions. **(A)** Empirical circular standard deviation of mid-reach angles in VIS + VIB trials versus minimum of VIS or VIB trials. Color reflects uncertainty level and marker style cue modality with the lowest reach variability for each participant (*N* = 31, point: VIS, triangle: VIB). **(B)** Mean mid-reach angle standard deviation and model predictions for different uncertainty levels. **(C)** Mean visual weights and model prediction for different uncertainty levels. Data in dark green and model predictions in blue. Error bars and shaded areas depict 95% confidence intervals. *p < 0.05, **p < 0.01, ***p < 0.001 for paired z-tests

We calculated the circular standard deviation of the mid-reach angles to further investigate the integration of visual and vibrotactile cues in estimating target location. We first tested if the reach variance in VIS + VIB trials was different from the individually determined lowest variance of the unimodal estimates (Fig. 4A). Fitting model 5a, we found that the full model fitted the data significantly better than the reduced model (likelihood-ratio test, χ^2^_(4)_ = 40.96, *p* < 0.001). Next, we found a significant interaction effect between uncertainty level and type (likelihood-ratio test, χ^2^_(3)_ = 35.98, *p* < 0.001). Using post-hoc tests, we found that only for the highest tested uncertainty level (60°), bimodal reach variability was lower than the lowest unimodal variability (paired z-test, z = 6.53, *p* < 0.001). There was no difference for all other uncertainty levels (paired z-test, 0°: z = − 1.67, *p* = 0.10; 15°: z = − 1.89, *p* = 0.06, 30°: z = 0.77, *p* = 0.44).

Next, we compared our data to MLE predictions. If integration follows MLE, the bimodal reach variability can be derived from the unimodal variances. Additionally, we removed movement-associated variability from our predictions (s. Methods, Eq. 2). Figure 4B shows the mid-reach standard deviations as a function of visual uncertainty of the observed data and MLE prediction. Fitting model 6a to our data, we found a significant difference between the full and reduced model (likelihood-ratio test, χ^2^_(4)_ = 4.69, *p* = 0.20). Additionally, we did not find a significant interaction effect of uncertainty and type, but significant main effects of both uncertainty (likelihood-ratio test, χ^2^_(3)_ = 343.5, *p* < 0.001) and type (likelihood-ratio test, χ^2^_(1)_ = 26.65, *p* < 0.001). Post-hoc test showed that the bimodal variance was higher than the one predicted by MLE for all uncertainty levels but 0° (paired z-test, 0°: z = − 1.64, *p* = 0.10; 15°: z = − 3.29, *p* = 0.001; 30°: z = − 2.33, *p* = 0.02; 60°: z = − 6.23, *p* < 0.001).

Similarly, we computed the visual weights for the different uncertainty levels to determine how much participants relied on visual information (Fig. 4C). Again, we found a significant difference between the full and reduced model 7a (likelihood-ratio test, χ^2^_(4)_ = 29.81, *p* = 0.20). We observed a significant interaction effect of uncertainty and type (likelihood-ratio test, χ^2^_(3)_ = 9.01, *p* = 0.04). Using post-hoc tests, we found lower visual weights than predicted by MLE once visual target uncertainty is introduced but not at 0° uncertainty (paired z-test, 0°: z = 1.84, *p* = 0.07; 15°: z = 3.60, *p* = 0.003; 30°: z = 2.40, *p* = 0.02; 60°: z = 5.25, *p* < 0.001). Results of model fits can be found in Table S2.

**Fig. 5:**
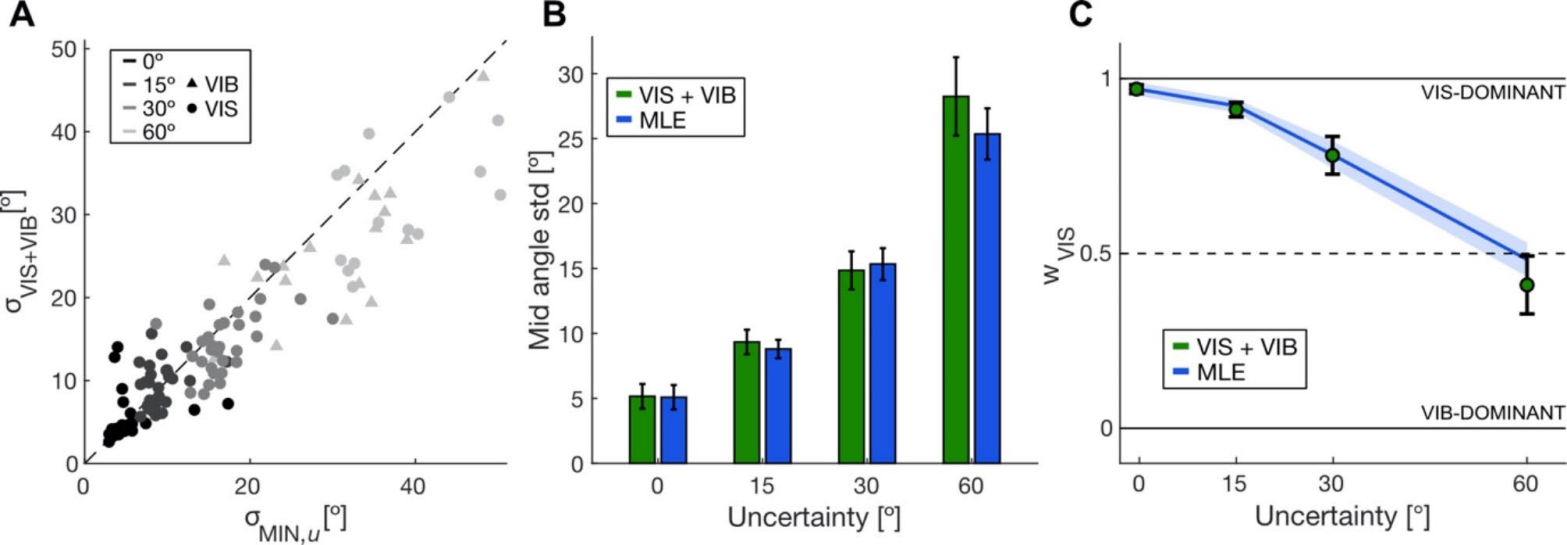
MLE predictions including only cardinal and oblique trials. **(A)** Empirical circular standard deviation of mid-reach angles in VIS + VIB trials versus minimum of VIS or VIB trials. Color reflects uncertainty level and marker style cue modality with the lowest reach variability for each participant (*N* = 31, point: VIS, triangle: VIB). **(B)** Mean mid-reach angle standard deviation and model predictions for different uncertainty levels. **(C)** Mean visual weights and model prediction for different uncertainty levels. Data in dark green and model predictions in blue. Error bars and shaded areas depict 95% confidence intervals. *p < 0.05, **p < 0.01, ***p < 0.001 for paired z-tests

Since we observed deficient performance for the intermediate directions, we wondered if MLE predictions were violated because participants were unable to interpret these cues correctly. Therefore, we ran the same analyses but excluded trials with intermediate targets. The behavioral results were similar to results including all directions and mainly differed in effect sizes (Fig. S1, Table S3). First we again tested if the empirical bimodal variability was different from the lowest individually determined unimodal variability. We found a significant difference between the full and reduced model when fitting model 5b to trials with only cardinal and oblique targets (Fig. 5A, likelihood-ratio test, χ^2^_(4)_ = 31.04, *p* < 0.001). Further, the interaction between uncertainty and type was significant and post-hoc tests showed lower bimodal variance compared to the lowest unimodal variability for high uncertainty levels (paired z-test, 30°: z = 3.86, *p* = 0.001; 60°: z = 4.56, *p* < 0.001). There was no difference for low uncertainty levels (paired z-test, 0°: z = 0.08, *p* = 0.94; 15°: z = − 0.49, *p* = 0.63).

Next, we tested if the empirical bimodal variability differed from the MLE prediction when trials with intermediate targets were excluded (Fig. 5B). Fitting model 6b, we did not find a significant difference between the full and reduced model (likelihood-ratio test, χ^2^_(4)_ = 9.04, *p* = 0.06) when including only cardinal and oblique target directions. Since the effect of type (observed data or model prediction) was not included in the reduced model, we did not find a difference between predicted and measured reach variabilities.

Similarly, we computed the visual weights for the different uncertainty levels to determine how much participants relied on visual information (Fig. 5C). Again, we found no significant difference between the full and reduced model 7b (likelihood-ratio test, χ^2^_(4)_ = 7.14, *p* = 0.13). Again, we did not find a significant difference between MLE predictions and empirical data. Detailed model results can be found in Table S4.

## Discussion

We tested how humans integrate visual and artificial vibrotactile reach instructions. We found that (i) participants could interpret vibration stimuli to estimate target locations; (ii) vibrotactile stimulation complementing visual information partly compensated for the uncertain visual information, particularly in cases of high visual target uncertainty, as reflected in higher hit rates and lower mid-reach errors; and (iii) participants integrated visual and vibrotactile cues following MLE predictions when targets were cued in cardinal and oblique directions. When participants needed to reach towards targets in intermediate directions, they still relied on vibrotactile inputs, but the integration no longer complied with MLE predictions. Additionally, we found reduced reaction times when vibrotactile cues complemented visual cues of low uncertainty. Our results show that participants benefitted from combining visual and vibrotactile cues without a need for extended training, highlighting the usefulness of such artificial sensory inputs.

### Integration of visual vibrotactile cues depends on stimulation patterns

Cue integration following MLE requires knowledge about the statistical properties of the sensory cues and the corresponding sensory system. Participants seemed to acquire this knowledge only in cases where targets were cued with one motor or two motors active with the same intensity. However, when targets were located in intermediate directions and the activity of two simultaneous vibrotactile cues had to be discriminated, participants did not weigh visual and vibrotactile inputs following MLE. Suboptimal integration here is likely due to the applied stimulation protocol causing errors in interpreting the vibrotactile cues rather than related to the integration process itself. This interpretation is supported by the strong performance differences between target directions. Errors in discriminating which motor had the higher intensity could have led to biases away from the actual target location, resulting in higher reach variance. As discussed in the next session, one reason might be the inherent complexity of this additional discrimination of two vibrotactile cues and the training protocol.

At the same time, we found evidence for nearly optimal integration of visual and artificial vibrotactile cues to determine the location of a reach target if it was located in cardinal or oblique directions. This finding aligns with previous research showing optimal integration across various sensory modalities [23–27]. We found reduced variability of target estimates in the bimodal condition compared to the individually determined lowest variability in the unimodal condition for high visual uncertainty levels. Additionally, bimodal estimates of the target location were not only more precise but also more accurate, reflected in higher hit rates and smaller reach errors in VIS + VIB compared to VIS or VIB trials.

Considering the comparable short training participants received, the results show that participants can quickly adopt strategies to integrate artificial vibrotactile inputs. In a different study, participants could learn to integrate arbitrary visual and touch signals by their statistical co-occurrence [43]. There, participants learned these associations over the time course of multiple sessions. In our experiment, they only had around 30 min to learn to associate visual with vibrotactile cues. Perhaps, additional learning could improve interpretation of intermediate target cues resulting in variabilities closer to the one predicted by MLE.

Moreover, despite suboptimal integration for intermediate directions, we found differences in reaction times but no effect of additional vibrotactile cues on movement times. This suggests that target uncertainty and integration primarily affect the target selection process during movement planning. While participants benefitted from additional vibrotactile cues regarding hit rate and reach error, particularly in high-uncertainty trials, we observed the opposite for reaction times. Here, participants reacted faster following bimodal cues only if the uncertainty level was low. Similarly, it has been shown that reaction times for visually-guided saccades were lower when additional auditory cues were present [44]. However, this previous study also found that reaction times were reduced less if the spatial discrepancy between the two cues increased. In the case of high visual target uncertainty, the unimodal estimated target locations are likely to be further apart in our experiment. This difference could have led to conflicting signals that needed to be resolved, resulting in similar reaction times to VIS trials. Indeed, it has been shown that such cross-modal congruency effects (CCE) can lead to shorter reaction times when visual and vibrotactile cues are spatially congruent [45, 46] and located within or outside of the peripersonal space [47]. The observed CCE suggests that while we did not find effects of multisensory integration on accuracy and precision when uncertainty was low (likely because visual cues alone were sufficient), participants still integrated additional vibrotactile inputs. Taken together, this means that participants always benefitted from vibrotactile cues, either in terms of accuracy when visual information was poor or speed when visual information was reliable.

In this study, we tested how visual and vibrotactile cues are integrated while participants had considerable time to combine the two signals and plan a reach. Future studies need to investigate how these results generalize to active movement control based on visual and vibrotactile feedback of hand position.

### Different performance in VIB trials depending on targets and high variability between participants

With the vibrotactile stimulation protocol presented here, we found that participants were able to use vibrotactile cues to estimate the location of a reaching target. However, there were substantial differences in how well participants could estimate a cued target depending on its direction. There are multiple potential reasons. First, due to the training protocol, individual cardinal targets were repeated more frequently than oblique targets, which, in turn, were repeated more frequently than targets in intermediate directions (see Methods section). Participants, therefore, had more repetitions to learn the vibrotactile mapping for cardinal and oblique targets. Hence, the performance in the different direction categories strongly correlates with the number of training trials. Second, for oblique and intermediate targets, the activation of two motors needed to be detected at the same time. Finally, for intermediated targets, the activation of two active motors had to be compared to determine which one vibrated stronger.

Moreover, previous studies in which only vibrotactile feedback was provided during reaching movements showed that participants tended to decompose their movements into sub-movements along cardinal directions [18, 48], suggesting that participants were unable to follow intermediate directions. These findings have been related to either the inability to form an appropriate vibrotactile map, insufficient attentional capacities, or the effect of masking, which describes the reduced sensitivity to a stimulus in the presence of a second stimulus [48]. These possible mechanisms might have led to improper weighing of vibrotactile inputs for intermediate target directions, which could have led to violations of MLE predictions. Potential ways to improve discrimination could be stimulating two sites not simultaneously but sequentially or placing the motors that need to be compared within the same dermatome [49, 50].

We additionally observed a high variability in performance between participants. One reason might be that the placement of the motors for individual participants was not optimized. As the diameter of the arm changes between participants, the motors at the lower arm might have been too close for some of them. Hence, the propagating vibration across the skin could have led to confusion about which motor was active [51].

### Limitations and alternative models of multisensory integration

Determining the predictions by MLE, we simplified the model by neglecting participants’ prior expectations and proprioceptive estimates about possible target locations. Since we had discrete target locations, those could have influenced the final estimate [52] and might explain the slight differences between the data and the model for high uncertainty conditions, where we found a bias towards vibrotactile cues, and these prior expectations potentially have the most substantial influence. Another reason for the observed bias in high uncertainty trials might be that participants partly disregarded visual inputs as it was too challenging to interpret visual cues with high uncertainty levels.

In this study, we focused on the reduction of variability resulting from integrating two sensory inputs. However, MLE makes predictions not only for the bimodal variance but also for the estimate itself. Here, we have not tested if the estimated target location in bimodal trials was consistent with MLE predictions. This could provide further evidence of MLE integration but would require conflict conditions not included in this study (e.g., [28]). Furthermore, we assumed that vibrotactile variability remained constant throughout the experiment and across sessions. To prevent additional learning of the vibrotactile mapping, we did not disclose the success of a trial or the actual target location. However, in cases of low visual uncertainty, congruent bimodal inputs could, in principle, have triggered learning processes, potentially altering vibrotactile variability and affecting MLE predictions. Nonetheless, we expect these influences to be small because directions and uncertainty levels were randomly interleaved within each vibrotactile block, preventing continuous learning signals.

Previous studies investigating multisensory integration have predominantly used psychometric methods to test MLE hypotheses. Our study used a motor task paradigm, which introduces a different type of noise in the behavioral readout, such as motor planning and execution noise [53, 54]. We accounted for motor variability by removing it from our model [38]. We consider other factors, like proprioceptive noise, negligible given the veridical visual cursor feedback about the movement.

Recently, a growing body of literature has raised caution against overinterpreting results from previous studies demonstrating MLE integration [31, 32] and argued against MLE as a general mechanism by finding evidence of suboptimal integration of sensory cues [29, 30]. To rule out that participants used the most reliable unimodal information, which is referred to as cue-switching [21], we determined the most reliable cue for each participant individually. We then compared it to the observed bimodal variance, as suggested previously [31]. Since the bimodal variability was lower than the minimum variability in VIS or VIB trials when visual uncertainty was high, participants showed increased precision in VIS + VIB trials, the central MLE prediction [55]. In cases of low visual uncertainty, it is likely that our method is not sensitive enough to detect small gains in precision. However, we note that alternative models, such as probabilistic cue-switching, can lead to predictions similar to MLE [30, 31]. Future experiments may disentangle those theories by matching the associated individual variances of the inputs to increase the sensitivity for differences in their predictions.

### Considerations for application

Here, we only observed near-optimal integration when the interpretation of vibrotactile information was based on detecting active motors. Consequently, future stimulation protocols seem favorable that do not require discriminating the intensities of two vibrotactile cues. Statistically optimal integration of visual and artificial vibrotactile information has an advantage compared to other suboptimal strategies: participants are likely to benefit from the additional vibrotactile information even if the stimulation paradigm is insufficient to achieve performance levels similar to purely visual ones, as we have seen in our experiment. By integrating and weighting the two inputs by their respective variance, the final bimodal estimate will be more accurate and have lower variance than movements solely relying on visual cues alone. Nonetheless, longer training durations with vibrotactile stimulation are likely to improve performance further. As shown in a recent study exposing participants to vibrotactile feedback over 20 sessions, resulting in accuracies comparable to purely proprioception-based reaches [48].

While most participants in our study were relatively young, the prevalence of neurological diseases increases with advancing age. Consequently, the demand for supplementary feedback becomes more critical among older cohorts. Interestingly, it has been shown that the ability to integrate information from different sensory sources varies during a person’s lifetime and seems to be enhanced in older adults (see 56 for a review). Thus, older patients may exhibit an even greater potential for benefitting from additional sensory inputs. However, it is worth noting that studies have shown vibration intensity discrimination to be worse in older people than in young adults while suggesting the potential for improvement through targeted training [48, 50].

## Conclusion

To conclude, participants can use vibrotactile cues to estimate the location of a reach target and benefit from vibrotactile cues when visual information is uncertain. Participants can adopt near-optimal strategies for vibrotactile cues after short training. However, due to the choice of vibrotactile stimulation, integration violated MLE prediction when vibration intensities needed to be discriminated. Therefore, patients provided with additional vibrotactile information are likely to quickly benefit from such stimulation when combined with uncertain visual inputs, even if the additional artificial input on its own might not lead to optimal performance levels. This rapid adaptability of beneficial integration strategies further highlights the usefulness and effectiveness of supplementary vibrotactile input.

## Electronic supplementary material

Below is the link to the electronic supplementary material.

Supplementary Material 1

Supplementary Material 2

## Data Availability

Data used to create the figures and perform statistical analyses can be found at GRO.data repository of the University of Göttingen, 10.25625/F5KYUT.

## Abbreviations

ANOVA: Analysis of variance
BCI: Brain–computer interface
CCE: Cross–modal congruency effects
EMM: estimated marginal mean
ERM motor: Eccentric rotating mass motor
GLMM: Generalized linear mixed–effect model
MLE: Maximum likelihood estimation
VIB: Trials with vibrotactile cues
VIS: Trials with visual cues
VIS + VIB: Trials with visual and vibrotactile cues
