# Supplementary material for: Optimality of multisensory integration while compensating for uncertain visual target information with artificial vibrotactile cues during reach planning
Source: J Neuroeng Rehabil. 2024 Sep 9;21:155. doi: 10.1186/s12984-024-01448-0 (PMC11382450; doi:10.1186/s12984-024-01448-0)
Supplement: Supplementary file 1 — Supplementary Material 1 [file 12984_2024_1448_MOESM1_ESM.pdf]

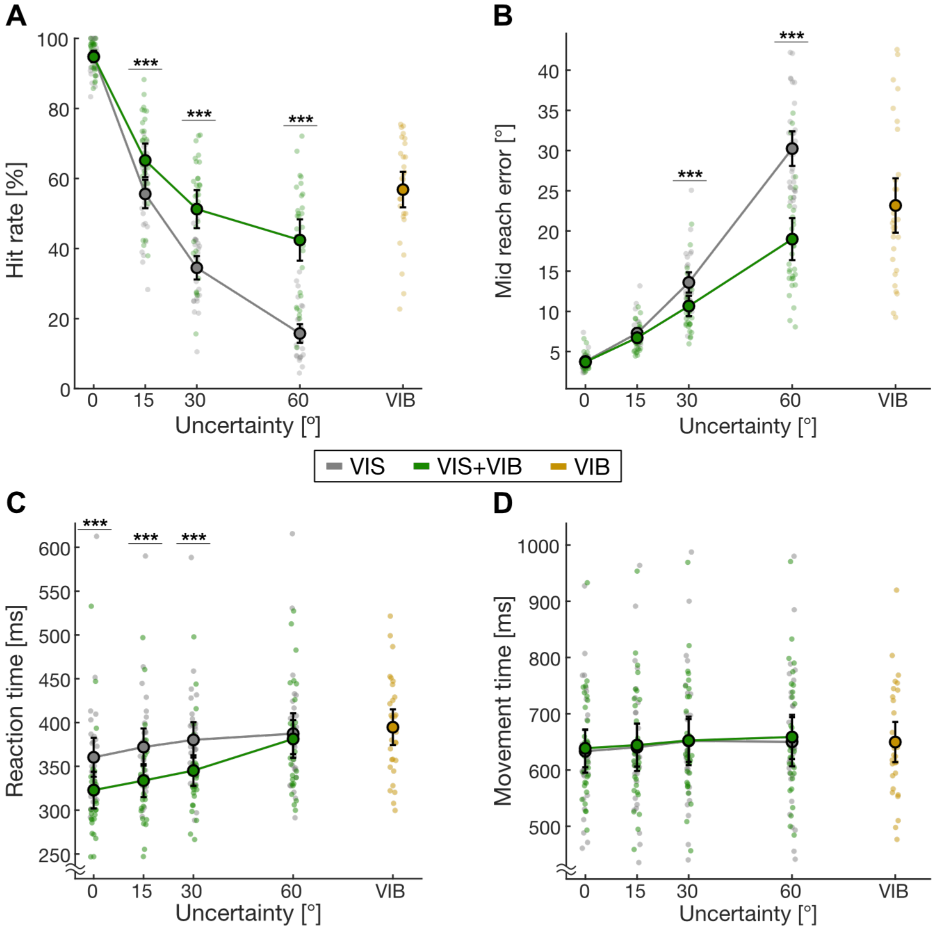

Fig. S1: Participants' performance dependent on uncertainty level and cue modality including only cardinal and oblique directions. A. Mean hit rate. **B.** Mean mid-reach reach error. **C.** Mean reaction time. **D**. Mean movement time. Results from VIS trials in grey, VIS+VIB trials in green, and VIB-only trials in yellow. Shaded points show data from individual participants (N = 31). Error bars depict 95% confidence intervals. *p<0.05, **p<0.01, ***p<0.001 for paired z-tests.
