# Supplementary material for: Optimality of multisensory integration while compensating for uncertain visual target information with artificial vibrotactile cues during reach planning
Source: J Neuroeng Rehabil. 2024 Sep 9;21:155. doi: 10.1186/s12984-024-01448-0 (PMC11382450; doi:10.1186/s12984-024-01448-0)
Supplement: Supplementary file 2 — Supplementary Material 2 [file 12984_2024_1448_MOESM2_ESM.pdf]

Table S1: GLMM model results for behavioral parameters including all directions.

Predictor	Estimate	Error	2.5% CL	97.5% CL	Df	Chisq	p	Min	Max
**Model 1a: Hit rate**									
Intercept	2.746	0.135	2.575	2.918				2.709	2.797
Uncertainty15	-2.656	0.123	-2.822	-2.494				-2.701	-2.609
Uncertainty30	-3.666	0.119	-3.837	-3.508				-3.704	-3.628
Uncertainty60	-4.651	0.134	-4.830	-4.478				-4.702	-4.610
Cue	-0.212	0.158	-0.398	-0.045				-0.247	-0.135
Uncertainty15 x Cue	0.303	0.164	0.101	0.503				0.229	0.346
Uncertainty30 x Cue	0.658	0.162	0.448	0.870				0.574	0.703
Uncertainty60 x Cue	1.211	0.181	0.974	1.439				1.120	1.262
Uncertainty x Cue					3	75.17	<0.001		
**Model 2a: Mid-reach error**									
Intercept	-3.174	0.027	-3.223	-3.122				-3.184	-3.165
Uncertainty15	0.368	0.021	0.326	0.410				0.358	0.375
Uncertainty30	0.781	0.024	0.731	0.829				0.773	0.787
Uncertainty60	1.388	0.034	1.323	1.451				1.380	1.399
Cue	0.006	0.026	-0.043	0.061				-0.002	0.013
Uncertainty15 x Cue	-0.016	0.029	-0.075	0.041				-0.026	-0.006
Uncertainty30 x Cue	-0.131	0.029	-0.188	-0.075				-0.143	-0.120
Uncertainty60 x Cue	-0.428	0.036	-0.495	-0.362				-0.443	-0.416
Uncertainty x Cue					3	77.52	<0.001		
**Model 3a: Reaction times**									
Intercept	-0.566	0.042	-0.651	-0.484				-0.595	-0.553
Uncertainty15	0.044	0.012	0.020	0.068				0.039	0.050
Uncertainty30	0.089	0.016	0.059	0.121				0.079	0.096
Uncertainty60	0.111	0.021	0.071	0.151				0.099	0.120
Cue	-0.172	0.035	-0.236	-0.106				-0.195	-0.161
Uncertainty15 x Cue	0.024	0.017	-0.011	0.056				0.017	0.037
Uncertainty30 x Cue	0.057	0.018	0.021	0.092				0.048	0.068
Uncertainty60 x Cue	0.190	0.021	0.149	0.227				0.183	0.208
Uncertainty x Cue					3	47.80	<0.001		
**Model 4a: Movement times**									
Intercept	0.577	0.090	0.407	0.748				0.526	0.602
Uncertainty15	0.018	0.019	-0.017	0.054				0.008	0.023
Uncertainty30	0.029	0.018	-0.006	0.061				0.022	0.035
Uncertainty60	0.050	0.019	0.013	0.086				0.044	0.056
Cue	0.027	0.025	-0.022	0.072				0.022	0.041
Uncertainty15 x Cue	0.017	0.016	-0.015	0.048				0.007	0.024
Uncertainty30 x Cue	0.027	0.016	-0.003	0.056				0.017	0.036
Uncertainty60 x Cue	0.036	0.019	0.001	0.075				0.022	0.043
Uncertainty x Cue					3	4.74	0.19		

Estimate corresponds to mean value. Error for standard error. Lower (2.5%) and upper (97.5%) confidence levels (CL) from bootstrap estimation (N=1000). Degrees of freedom (Df), Chi-square value (Chisq), and p-value from likelihood ratio test. Minimum and maximum value from model stability results. Cue predictor had VIS cue condition as reference.

Table S2: GLMM model results for MLE predictions including all directions.

Predictor	Estimate	Error	2.5% CL	97.5% CL	Df	Chisq	p	Min	Max
**Model 5a: Minimum variance**									
Intercept	-5.540	0.137	-5.845	-5.290				-5.613	-5.515
Uncertainty15	1.299	0.137	1.042	1.581				1.234	1.362
Uncertainty30	2.542	0.127	2.298	2.838				2.487	2.618
Uncertainty60	4.013	0.129	3.783	4.306				3.965	4.087
Type	0.270	0.162	-0.052	0.603				0.206	0.325
Uncertainty15 x Type	-0.004	0.182	-0.366	0.348				-0.066	0.067
Uncertainty30 x Type	-0.320	0.168	-0.665	0.049				-0.375	-0.266
Uncertainty60 x Type	-0.617	0.165	-0.965	-0.272				-0.671	-0.552
Uncertainty x Type					3	35.98	<0.001		
**Model 6a: MLE variance**									
Intercept	-5.539	0.132	-5.841	-5.320				-5.603	-5.514
Uncertainty15	1.256	0.134	1.016	1.555				1.204	1.311
Uncertainty30	2.350	0.124	2.136	2.624				2.310	2.417
Uncertainty60	3.378	0.125	3.161	3.665				3.348	3.447
Type	0.257	0.156	-0.055	0.581				0.192	0.305
Uncertainty15 x Type	0.049	0.178	-0.316	0.399				-0.009	0.123
Uncertainty30 x Type	-0.114	0.164	-0.453	0.234				-0.160	-0.058
Uncertainty60 x Type	0.035	0.161	-0.300	0.365				-0.016	0.099
Uncertainty x Type					3	4.89	0.20		
**Model 7a: Visual weights**									
Intercept	4.036	0.141	3.785	4.353				4.015	4.136
Uncertainty15	-1.313	0.138	-1.622	-1.067				-1.394	-1.275
Uncertainty30	-2.492	0.129	-2.787	-2.248				-2.587	-2.455
Uncertainty60	-3.904	0.136	-4.212	-3.675				-3.997	-3.876
Type	-0.302	0.164	-0.619	0.017				-0.371	-0.218
Uncertainty15 x Type	-0.071	0.182	-0.448	0.282				-0.12	-0.013
Uncertainty30 x Type	0.115	0.17	-0.227	0.463				0.041	0.185
Uncertainty60 x Type	-0.177	0.185	-0.547	0.191				-0.26	-0.094
Uncertainty x Type					3	9.01	0.04		

Estimate corresponds to mean value. Error for standard error. Lower (2.5%) and upper (97.5%) confidence levels (CL) from bootstrap estimation (N=1000). Degrees of freedom (Df), Chi-square value (Chisq), and p-value from likelihood ratio test. Minimum and maximum value from model stability results. Type predictor had minimum unimodal variance or MLE prediction as reference.

Table S3: GLMM model results for behavioral parameters without intermediate directions.

Predictor	Estimate	Error	2.5% CL	97.5% CL	Df	Chisq	p	Min	Max
**Model 1b: Hit rate**									
Intercept	2.992	0.135	2.749	3.263				2.952	3.055
Uncertainty15	-2.762	0.123	-3.020	-2.539				-2.826	-2.709
Uncertainty30	-3.670	0.119	-3.919	-3.455				-3.718	-3.619
Uncertainty60	-4.738	0.134	-5.020	-4.472				-4.809	-4.686
Cue	-0.026	0.158	-0.333	0.305				-0.107	0.046
Uncertainty15 x Cue	0.460	0.164	0.124	0.768				0.387	0.543
Uncertainty30 x Cue	0.756	0.162	0.432	1.066				0.674	0.841
Uncertainty60 x Cue	1.432	0.181	1.063	1.793				1.337	1.515
Uncertainty x Cue					3	52.74	<0.001		
**Model 2b: Mid-reach error**									
Intercept	-3.170	0.040	-3.240	-3.104				-3.179	-3.158
Uncertainty15	0.351	0.016	0.285	0.409				0.334	0.358
Uncertainty30	0.719	0.020	0.656	0.781				0.708	0.730
Uncertainty60	1.367	0.024	1.273	1.456				1.354	1.379
Cue	-0.009	0.032	-0.080	0.064				-0.02	-0.001
Uncertainty15 x Cue	-0.086	0.022	-0.162	0.005				-0.103	-0.072
Uncertainty30 x Cue	-0.233	0.024	-0.312	-0.156				-0.257	-0.214
Uncertainty60 x Cue	-0.637	0.028	-0.734	-0.537				-0.659	-0.614
Uncertainty x Cue					3	78.73	<0.001		
**Model 3b: Reaction times**									
Intercept	-0.570	0.040	-0.647	-0.492				-0.598	-0.558
Uncertainty15	0.041	0.016	0.010	0.073				0.032	0.046
Uncertainty30	0.086	0.020	0.046	0.125				0.074	0.095
Uncertainty60	0.105	0.024	0.057	0.156				0.084	0.112
Cue	-0.173	0.032	-0.235	-0.111				-0.185	-0.159
Uncertainty15 x Cue	0.005	0.022	-0.038	0.051				0.000	0.022
Uncertainty30 x Cue	0.006	0.024	-0.042	0.051				-0.007	0.018
Uncertainty60 x Cue	0.142	0.028	0.087	0.193				0.132	0.164
Uncertainty x Cue					3	22.05	<0.001		
**Model 4b: Movement times**									
Intercept	0.566	0.089	0.389	0.739				0.515	0.591
Uncertainty15	0.020	0.017	-0.014	0.050				0.013	0.023
Uncertainty30	0.043	0.018	0.009	0.079				0.036	0.052
Uncertainty60	0.062	0.020	0.021	0.101				0.055	0.069
Cue	0.016	0.028	-0.041	0.071				0.010	0.034
Uncertainty15 x Cue	-0.002	0.021	-0.040	0.040				-0.010	0.006
Uncertainty30 x Cue	-0.008	0.021	-0.047	0.033				-0.016	0.001
Uncertainty60 x Cue	0.018	0.025	-0.032	0.068				0.005	0.027
Uncertainty x Cue					3	1.01	0.82		

Estimate corresponds to mean value. Error for standard error. Lower (2.5%) and upper (97.5%) confidence levels (CL) from bootstrap estimation (N=1000). Degrees of freedom (Df), Chi-square value (Chisq), and p-value from likelihood ratio test. Minimum and maximum value from model stability results. Cue predictor had VIS cue condition as reference.

Table S4: GLMM model results for MLE predictions without intermediate directions.

Predictor	Estimate	Error	2.5% CL	97.5% CL	Df	Chisq	p	Min	Max
**Model 5b: Minimum variance**									
Intercept	-5.415	0.144	-5.722	-5.184				-5.558	-5.386
Uncertainty15	1.080	0.145	0.791	1.365				0.996	1.191
Uncertainty30	2.333	0.137	2.079	2.620				2.272	2.472
Uncertainty60	3.699	0.154	3.431	4.013				3.641	3.853
Type	-0.013	0.175	-0.392	0.372				-0.090	0.045
Uncertainty15 x Type	0.070	0.201	-0.344	0.491				0.020	0.139
Uncertainty30 x Type	-0.292	0.183	-0.694	0.075				-0.340	-0.217
Uncertainty60 x Type	-0.377	0.197	-0.772	0.053				-0.446	-0.293
Uncertainty x Type					3	11.95	0.007		
**Model 6b: MLE variance**									
Intercept	-5.443	0.139	-5.760	-5.190				-5.556	-5.410
Uncertainty15	1.047	0.141	0.783	1.372				0.969	1.133
Uncertainty30	2.120	0.131	1.888	2.408				2.065	2.232
Uncertainty60	3.130	0.143	2.867	3.443				3.084	3.247
Type	-0.018	0.170	-0.354	0.326				-0.097	0.027
Uncertainty15 x Type	0.125	0.196	-0.242	0.536				0.083	0.198
Uncertainty30 x Type	-0.046	0.179	-0.406	0.329				-0.098	0.034
Uncertainty60 x Type	0.233	0.185	-0.111	0.610				0.184	0.318
Uncertainty x Type					3	6.87	0.08		
**Model 7b: Visual weights**									
Intercept	3.612	0.160	3.336	3.997				3.585	3.850
Uncertainty15	-1.064	0.145	-1.383	-0.772				-1.261	-1.007
Uncertainty30	-2.250	0.146	-2.573	-1.976				-2.472	-2.192
Uncertainty60	-3.763	0.177	-4.157	-3.448				-3.976	-3.719
Type	0.049	0.181	-0.304	0.419				-0.061	0.126
Uncertainty15 x Type	-0.171	0.201	-0.570	0.229				-0.235	-0.057
Uncertainty30 x Type	-0.034	0.189	-0.398	0.316				-0.105	0.065
Uncertainty60 x Type	-0.435	0.253	-0.994	0.058				-0.535	-0.297
Uncertainty x Type					3	4.39	0.22		

Estimate corresponds to mean value. Error for standard error. Lower (2.5%) and upper (97.5%) confidence levels (CL) from bootstrap estimation (N=1000). Degrees of freedom (Df), Chi-square value (Chisq), and p-value from likelihood ratio test. Minimum and maximum value from model stability results. Type predictor had minimum unimodal variance or MLE prediction as reference.
